# Zinc finger E-Box binding homeobox 2 (ZEB2)-induced astrogliosis protected neuron from pyroptosis in cerebral ischemia and reperfusion injury

**DOI:** 10.1080/21655979.2021.2012551

**Published:** 2021-12-13

**Authors:** Zhixin Zhao, Xiaoming Hu, Jie Wang, Jianfeng Wang, Yong Hou, Suyun Chen

**Affiliations:** a Department of Neurosurgery, Taizhou Hospital of Zhejiang Province Affiliated to Wenzhou Medical University, Zhejiang Province, China; b Department of Clinical Laboratory, Taizhou Hospital of Zhejiang Province Affiliated to Wenzhou Medical University, Zhejiang Province, China

**Keywords:** ZEB2, pyroptosis, Mcao/R, astrocyte, neural regeneration

## Abstract

Ischemia injury can cause cell death or impairment of neuron and astrocytes, and thus induce loss of nerve function. central nervous systems injury induces an aberrant activation of astrocytes called astrogliosis. Pyroptosis, which is a kind of programmed cell death, was proved play an important role in ischemia injury. Zinc Finger E-Box Binding Homeobox 2 (ZEB2) promoted neuron astrogliosis, which play a protected role in neuron regeneration. However, its precise mechanism remains unclear. This study investigated the mechanism of ZEB2 on astrogliosis and neuron regeneration after cerebral ischemia reperfusion condition. To confirm our hypothesis, Neurons and astrocytes were isolated from fetal Sprague Dawley rats, *in vivo* Middle Cerebral Artery Occlusion/reperfusion (MCAO/R) rat model and *in vitro* oxygen-glucose deprivation/reperfusion (OGD/R)-treated astrocytes and neurocytes model were constructed. Our results showed that ZEB2 was expressed in nucleus of astrocyte and upregulated after OGD/R induction, ZEB2 promoted astrogliosis. Further upregulation of ZEB2 promoted the astrogliosis, which promoted neuron proliferation and regeneration by decreased pyroptosis. Moreover, this finding was further confirmed *in vivo* MCAO/R rat model. Overexpression of ZEB2 promoted astrogliosis, which decreased infarct volume and accumulated recovery of neurological function by alleviated pyroptosis. This finding revealed that ZEB2 was a regulator of the astrogliosis after ischemia/reperfusion injury, and then astrogliosis promoted neuron regeneration via decreased neuron pyroptosis. Therefore, ZEB2 may be a potential therapeutic target for ischemia/reperfusion injury.

## Introduction

Stroke, including ischemic stroke and hemorrhagic stroke, is currently one of the leading causes of human disability and mortality worldwide, of which 87% are ischemic stroke caused by obstruction of the cerebral arteries [1,2]. When ischemic stroke occurs, impaired blood supply to the brain tissue, leading to ischemia and hypoxia, which in turn leads to clinical manifestations of the corresponding neurological deficits, thus resulting in disability, or even death of the patient after an ischemia stroke [3,4]. Recently, to release vascular occlusion and restore cerebral perfusion, intravenous thrombolysis and mechanical thrombectomy are the two main strategies to achieve cerebral blood flow recanalization [5]. However, both the clinical applications are restricted by a narrow therapeutic window (only 4.5–6 h of intravenous thrombolysis and no more than 24 h of mechanical thrombectomy). Therapy beyond the therapeutic window can create reperfusion injury and aggravate the degree of brain tissue damage [6]. Neuroprotective therapies, as an adjunctive therapies to revascularization therapy, can increase the survival of nerve cells and promote the recovery of nerve function [7]. However, there has still been no successful clinical translation of neuroprotective agents to promote neuronal survival in stroke patients [8,9]. Thus, it is urgent to uncover the mechanisms of neuroprotection and neuron regeneration which can be useful in the development of new treatment methods.

In the conventional knowledge, apoptosis accounts for a significant proportion of neuronal death after cerebral ischemia [10]. Recently, however, studies found that various cell death mechanisms, including pyroptosis, also play roles in cerebral ischemia injury [11,12]. Pyroptosis is a pro-inflammatory programmed cell death that mainly mediated by inflammasome of nucleotide oligomerization domain like receptors (NLRs) and depends on Caspase family [13]. It is manifested by cellular distension until the cell membrane ruptures, leading to the release of cellular contents and thus activating a strong inflammatory response [14]. According to the activation mechanism, pyroptosis can divided into caspase-1 dependent and non-caspase dependent pathway [15]. Both pathways are active by cleavage of Gasdermin-D (GSDMD), an effector of pyroptosis, and therefore allows the release of N-terminal pore-forming domain, which insert into the plasma membrane, forming pore that induce plasma membrane rupture and release of intracellular contents including interleukin (IL) – 1β and IL – 18 [16]. Poh et al. provided the evidence that the NLR-family CARD-containing protein 4 (NLRC4) inflammasome complex mediates the apoptosis and pyroptosis of microglial cells under ischemic conditions [17]. Liang et al. confirmed that inhibition of Triggering Receptor Expressed on Myeloid Cells 1 (TREM-1) protects against ischemia-induced neuronal damage and alleviates microglial mediated neuro-inflammation by reducing oxidative stress and pyroptosis which observed in neuron, astrocytes, and microglia after cerebral ischemia conditions [18]. However, little is known the precise role of pyroptosis in cerebral injury under ischemic conditions.

Astrocytes are an important component of the central nervous systems (CNS), serving as support and compartmentalization of nerve cells and participating in the formation of the blood-brain barrier [19]. Unlike the peripheral nervous system, axons fail to regenerate effectively after CNS injury in adult mammals, often resulting in irreversible loss of function [20,21]. Astrocytes play important regulatory roles in axonal regeneration during the pathophysiology of CNS injury [21]. When CNS injury occurs, it evokes a process of change in astrocyte, which is defined as astrogliosis, including processes of hypertrophy and thickening [22]. In order to protects the surrounding tissue from further damage, activated astrocytes adjacent to the lesion are tightly organized to forms a dense gelatinous scar around the wound, preventing the spread of inflammatory cells or pathogens to normal tissues and maintaining the homeostasis of the internal environment [23]. In addition, astrogliosis also promote blood-brain-barrier repair, attenuate osmotic and oxidative stresses, and thus promote axon regeneration [24]. Studies confirmed that disruption of the astrocyte responded to injury increases the size of the lesion and exacerbates the recovery of neurological function [25–27]. Prevailing evidence suggested that Zinc Finger E-Box Binding Homeobox 2 (ZEB2) is a significant factor in astrogliosis, the overexpression of ZEB2 can improve neuroprotection and recovery of neurological function in spinal cord injury and cerebral ischemic stroke by evoking astrogliosis [28]. ZEB2 is a zinc-finger homeodomain transcription factor protein belong to ZEB family, ZEB2 play an essential role in neuronal development [29], also participate in Epithelial-mesenchymal transition (EMT) of cancer cell [30–33], wound healing [34] and neural development in adult [29,35]. ZEB2 was found up-regulated in cerebral ischemia reperfusion injury rats, further increased expression of ZEB2 attenuated the cerebral ischemia reperfusion injury, whereas knockdown of ZEB2 increased lesion size of cerebral ischemia reperfusion [28]. However, the role of ZEB2 in neural regeneration after cerebral ischemia reperfusion is unknown.

In this study, we hypothesized that astrocyte-derived ZEB2 enhanced the astrogliosis and hence promoted neural regeneration. Therefore, our goal is to investigate the relationship between ZEB2 expression in astrocytes and neural regeneration *in vivo* and *in vitro*. We found that ZEB2 is a regulator of astrocytes activity, which can evoke subsequent neural regeneration by suppressing pyroptosis.

## Materials and methods

### Primary cell culture of neuron and astrocytes and plasmids transfected

Primary hippocampal neurons and astrocytes were isolated from Sprague Dawley (SD) rat hippocampus as described [36,37]. In brief, SD fetal rats at 18–20 days of gestation were selected as the source of primary hippocampal cell culture. The pregnant rats were anaesthetized with ether, and the fetuses were dissected and decapitated one by one. After decapitation, the whole brain tissue was stripped out in iced Hibernate-E solution and the hippocampus was stripped bilaterally under a microscope. Neurons were cultured on poly-D-lysine coated glass coverslips in NbActiv4 (BrainBits, USA) medium containing antibiotic-antimycotic at 37°C. Passage 5–7 were used for experiment. Astrocytes were cultivated in Basal Eagle medium containing 10% fetal bovine serum, 0.45% glucose, 1 mM sodium pyruvate, 2 mM glutamate supplement and antibiotic-antifungal agent. Passage 3–5 were used for experiment.

Cells were divided into 4 groups: Ctrl group (astrocytes and neurons were co-cultured under normal condition), oxygen-glucose deprivation/reoxygenation (OGD/R) group (astrocytes and neurons were co-cultured under Glucose-deficient anaerobic condition), OGD/R + negative control (NC) group (astrocytes were transfected with pcDNA3.1-NC and then co-cultured with neurons under Glucose-deficient anaerobic condition), and OGD/R + ZEB2 group (astrocytes were transfected with pcDNA3.1-ZEB2 and then co-cultured with neurons under Glucose-deficient anaerobic condition). AS for cell transfection, pcDNA3.1-ZEB2 and pcDNA3.1- NC were synthesized from Genomeditech (Shanghai, China). Astrocytes were cultured in normal dulbecco’s modified eagle medium (DMEM) medium (Sigma China) under normal condition, pcDNA3.1-ZEB2 and pcDNA3.1-NC were transfected in astrocytes, respectively. After 48 hours, astrocytes were collected and inoculated in upper chamber of transwell with glucose-free DMEM (Sigma China) and neurons were inoculated in lower chamber with glucose-free DMEM (Sigma China). And then transwell inserts were placed in an anaerobic chamber (95% N_2_, 5% CO_2_ and 1% O_2_) at 37°C for 4 hours. Thereafter, cell culture medium was replaced into normal DMEM medium under normal condition for 48 hours for reperfusion.

### Cell viability assay

Cell viability of astrocytes and neurons were detected by the Cell Counting Kit-8 (CCK8) (abcam, China) [38]. After OGD/R treatment, cells (1 × 10^4^ per well) were inoculated in 96-well plates with normal DMEM medium and 10 μL CCK8 solutions were added in each well for 2 hours at 37°C, then cell viability will determine by spectrometry at 450 nm after incubation.

### Cell apoptosis assay

Cell apoptosis of astrocytes and neurons was assessed using Annexin V-FITC Apoptosis Detection Kit (Sigma China) [39], according to the instruction of manufacturer. After stimulation with OGD/R condition, cells were inoculated in 6-well plates and 100 μL of 1 × 10^6^ cells/mL cell suspension were stained with 5 μL of Annexin V-FITC and 10 μL of Propidium iodide (PI) for 15 min at 37°C in dark, then the annexin V-FITC binding were assessed the flow cytometry at an excitation wavelength of 488 nm.

### Immunofluorescence staining [28]

Cells fixed in 4% paraformaldehyde for 15 min. After fixation, cells permeabilized with 0.3% triton X-100 for 30 min, blocked by 10% goat serum for 60 min, then incubated with primary antibodies 5-bromo-2ʹ-dexoyuridine (BrdU) (1:250, ab6326, abcam), Neuronal nuclei (NeuN) (1:1000, ab104224, abcam), glial fibrillary acidic protein (GFAP) (1:500, ab33922, abcam), ZEB2 (1:5000, bs-20,485 R, Bioss), brain-derived neurotrophic factor (BDNF) (1:500, ab108319, abcam) at 4°C overnight. After that, the cells were incubated with secondary antibody including Fluorescein at room temperature for 2 hours in dark. All nuclei were stained with 4′,6-diamidino-2-phenylindole (DAPI) for 2 minutes in dark. Image was performed on a fluorescence microscope.

### Intracerebroventricular injection

SD rats (grand SPF, weighing 240–270 g) were purchased from the department of experimental animals, Kunming Medical University (Yunnan, China). Adeno-associated virus (AAV) included GFAP – specific promoters were used to pack plasmids and synthesized by HanBio, China. Rats were fasted 12 hours before surgery, and anesthetized by 42 mg/kg sodium pentobarbital, and they were fixed on the stereotaxic apparatus after they completely unconscious and the hair was removed. An incision was made along with midline scalp to expose the bregma, 1 μL of AAV-ZEB2 or its negatived control vector (1 × 10^7^ TU/mL, at a rate of 0.5 μL/min) was injected into right lateral ventricle [40]. The vectors were injected 20 days prior to the established MCAO/R model. Stereotactic coordinates were as follows: Anteroposterior, 0.8 mm; Mediolateral, 1.5 mm; Depth, 3.5 mm.

### Middle cerebral artery occlusion-reperfusion (MCAO/R) model establishment

This laboratory animal ethics (KMMU2020502, 20,200,815) has been approved by the Kunming Medical University ethics committee. The experimental procedures strictly followed the guidelines for the management and the usage of laboratory animals formulated by the national institutes of health according to the previous studies [41,42].

All rats were divided into 3 groups: sham group (n = 6), MCAO/R group (n = 6) and MCAO/R + ZEB2 group (n = 6). In brief, rats were anesthetized by injection of sodium pentobarbital (42 mg/kg), and they were fixed on the stereotaxic apparatus after they have become completely unconscious. After hair removed, an incision was made along with midline cervical to exposed the right common carotid artery (CCA), internal carotid artery (ICA) and external carotid artery (ECA). CCA and ECA were tied off and ICA was closed. The monofilament suture was inserted from ECA and advanced into ICA. 2 hours after surgery, 24 hours reperfusion was performed by removing the monofilament suture. Sham rats were subjected to the same procedure without MCAO/R.

### Neurological function test

Neurological function was assessed on modified Neurological Severity Scores (mNSS) [43] and Morris water maze [44] on post-MCAO/R days 1, 3, 7, 14, 21 and 28 days. The mNSS is a composite test of motor, sensory systems, reflexes and balance, the scoring method follows the description [43]. The higher scores indicate more severe neurological impairment of CNS.

To assess the spatial learning and memory of rats, we performed Morris water maze (MWZ) test. The MWZ pool (140 cm diameter, 60 cm height) were split into four quadrants with a platform (9 cm diameter, 23 cm height), which was placed in the third quadrant. MWZ test were divide to three parts, visible platform test from day 1 to day 2, hidden platform test from day 3 to day 7, probe test on day 8. In visible platform test, rats were given 20 seconds to acclimatize on the platform and then placed in each quadrant separately and had 60 seconds to reach the platform. In hidden platform test, the platform was hidden 1 cm under water, and then rats were placed in each quadrant separately and had 60 seconds to reach the platform. In probe test, the platform was removed and the rats were placed to each quadrant for 60 seconds. The time and movement track of rats in MWZ test was recorded by Ethovision XT monitoring system (Noldus, China). The escape latency, swimming path, and target zone frequency were analyzed.

### Neurological regeneration test

Neurological regeneration was tested by Nissl staining and Luxol fast blue staining [45] according to the instruction of manufacture. Rats were anesthetized by 3% pentobarbital sodium (42 mg/kg), injection and intracardially perfused with paraformaldehyde. The brain tissues were isolated and fixed in 4% paraformaldehyde for 24 hours, after which the hippocampus and cerebral cortex of the brain tissue were isolated. The brain tissues were dehydrated with 30% sucrose solution at 4°C for 3–5 days. After that, the brain tissues were preserved in opti-mum cutting temperature compound (OCT) for 6 hours at room temperature. Then the brain tissues were sliced into 8 and 20 μm thick sections at −20°C, respectively. Luxol fast blue (LFB) staining can demonstrated myelin. Briefly, 8 μm coronal brain slices were placed in LFB solution (Solarbio, China) at 60°C for 4 hours, then transferred to Lithium Carbonate for 30 seconds, obtained the image under bright field microscope (Leica, Germany). Nissl staining can demonstrate Nissl bodies. Briefly, 20 μm coronal brain slices were dehydrated in graded alcohol, staining by 0.1% cresyl violet (Solarbio, China), dehydrated in graded alcohol and xylene again, then transfer to Nissl Differentiation Solution (Solarbio, China) for 30 seconds, obtained the image under bright field microscope (Leica, Germany).

### Enzyme linked immunosorbent assay (ELISA)

The secretion of pro-inflammatory cytokines IL −1β and IL-18 in cells and brain tissue were detected by ELISA kits (abcam, China) [46], respectively. As manufacturer’s instruction, collected the cells and the cells were centrifuge at 2000 g for 10 minutes, after that, the cell supernatant was collected. Brain tissue lysates were centrifuge at 18,000 g for 20 minutes, after that the supernatants were collected. 50 μL per well samples supernatant and 50 μL per well antibody cocktail were added in to 96 plate wells, and the incubated for 1 hours at room temperature sharked with 400 rpm. After washed with 1X Wash Buffer PT, 100 μL 3,3ʹ,5,5ʹ-tetramethylbenzidine (TMB) development solutions were added in each well and incubated for 10 minutes in the dark sharked with 400 rpm. 100 μL of stop solution were added in each well, sharked whit 1 min for mixed and then the secretion of IL-1β and IL-18 will determine by spectrometry at 450 nm after incubation.

### Infarct volume assessment

The infarct volume was analyzed by 2,3,5-triphenyltetrazolium chloride (TTC, Sigma China) staining [47]. After model establishment, rats were euthanized immediately after reperfusion, then brain was harvested and frozen for 5 minutes at −80°C. Brains were cut into 2 mm sections placed in 2% TTC solution for 30 minutes at 37°C in dark. The stained brain slices were arranged in the order, and then labeled, ruled and photographed, and the infarct volume was calculated using Image-Pro Plus image processing software (infarct volume = infarct area of each slice × 2 mm).

### Western blot assay

Rats were euthanized followed the guideline of Kunming medical university, and the brain tissue was isolated. The total protein of cells or brains tissue were extracted by RIPA buffer (abcam, China) with protease inhibitor cocktail (abcam, China). Pierce BCA protein assay (abcam, China) were performed to determine protein concentration. The protein sample were separated by sodium dodecyl sulfate-12 polyvinylidene gel (SDS-PAGE) electrophoresis and then transferred to polyvinylidene fluoride (PVDF) membranes [48]. The membranes were blocked with 5% nonfat milk in TBST for 1 hour at room temperature. Membranes were incubated with primary antibodies caspase-1 (1:100, ab74297, abcam), IL-1β (1:1000, ab205924, abcam), NLRP3 (1:1000, ab263899, abcam), gasdermin (1:1000, ab219800, abcam), cleaved N-terminal GSDMD (1:1000, ab215203, abcam) overnight at 4°C, respectively. The membranes were washed with TBST, then incubated with secondary antibody for 2 hours at room temperature. Membranes were visualized by enhanced chemoluminescence (ECL) system. GADPH was used as a loading control. The protein bands intensities were imaged using an Odyssey Fc Image System and protein intensities were normalized GADPH.

### Statistics

All data were performed using GraphPad Prism 9.0 and expressed as mean ± standard deviation ($\overset{ˉ}{x}$ ± SD), differences between two groups were analyzed using Student’s *t*-test, differences between three or more groups were analyzed by one-way ANOVA with a Tukey test. Differences with *p < 0.05* were considered a significantly differences [18].

## Results

### *ZEB2 prompted astrogliosis* in vitro *under OGD/R*

In this manuscript, we assumed that ZEB2 promoted astrogliosis and therefore promote neuron regeneration via decreased neuron pyroptosis. Therefore, we first verified the function of ZEB2 in inducing astrogliosis. Astrogliosis is accompanied by increased glial fibrillary acidic protein (GFAP) expression, thus, elevated expression of GFAP is a hallmark signal for activation of astrocyte [28,49].

**Figure 1.**
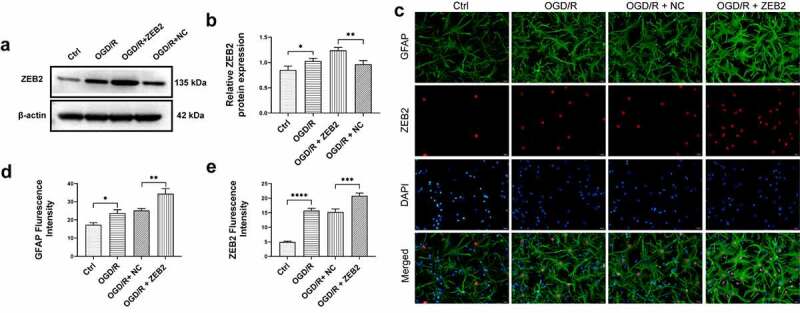
ZEB2 promoted the activation of astrocyte after OGD/R *in vitro.* A: Western blot assay of ZEB2 expression in astrocyte. B: Quantification of Western blot band intensity. C: Immunofluorescence staining for GFAP (green fluorescence), ZEB2 (red fluorescence), and DAPI (blue) in astrocytes. D: Fluorescence intensity of ZEB2. E: Fluorescence intensity of GFAP. E: Fluorescence intensity of ZEB2. E: Fluorescence intensity of GFAP. (Error bars represent mean ± SD, Magnification:400×; *p < 0.05, **p < 0.01, ***p < 0.001, ****p < 0.0001)

To evaluate expression of ZEB2 in astrocyte under OGD/R condition, Western blot assay was performed. The results demonstrated that protein expression of ZEB2 was significantly increased in OGD/R group compared with Ctrl group, and the expression of ZEB2 was further increased when transfected with pcDNA3.1 ZEB2, which indicated that the transfection of pcDNA3.1 ZEB2 was successful (Figure 1a, b). To further evaluate the subcellular localization and expression of ZEB2 in astrocytes, immunofluorescence assay was performed. As shown in Figure 1c-E, the astrocyte marker GFAP is mainly expressed in the cytoplasm, while ZEB2 is mainly expressed in the nucleus of astrocytes. In addition, the expression of ZEB2 and GFAP was significantly upregulated in OGD/R group compared to the control group, while a further increase of GFAP expression was observed when pcDNA3.1 ZEB2 was transfected. This result indicated that OGD/R induced astrogliosis, and the upregulation of ZEB2 further increase the activated astrocytes.

### ZEB2 protect neuron from pyroptosis via prompting astrogliosis

**Figure 2.**
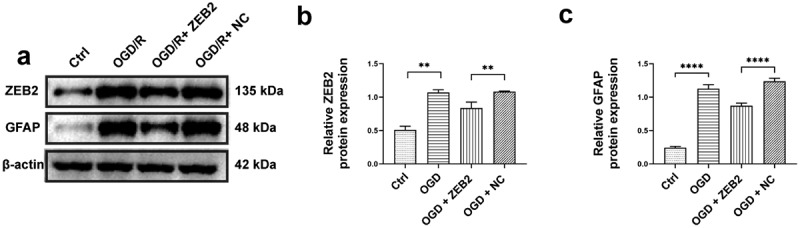
ZEB2 promoted astrogliosis after OGD/R in vitro. A: Western blot assay of ZEB2 and GFAP expression in astrocyte. B: Quantification of ZEB2 Western blot band intensity. C: Quantification of ZEB2 Western blot band intensity. (Error bars represent mean ± SD; *p < 0.05, **p < 0.01, ***p < 0.001, ****p < 0.0001).

Prevailing evidence suggested that ZEB2 mainly expressed in astrocytes of brain tissue in rats, and knockdown of ZEB2 *in vivo* suppressed neural regeneration [28]. To investigate the relationship between ZEB2 expression in astrogliosis and neural regeneration in *vitro*, we co-cultured astrocytes and neurons. The expression of GFAP and ZEB2 in co-cultured astrocytes were detected by western bolt, the results shown that the expression of ZEB2 and GFAP were increased under OGD/R condition, and the upregulation of ZEB2 further increased the expression of GFAP (Figure 2a-c).

**Figure 3.**
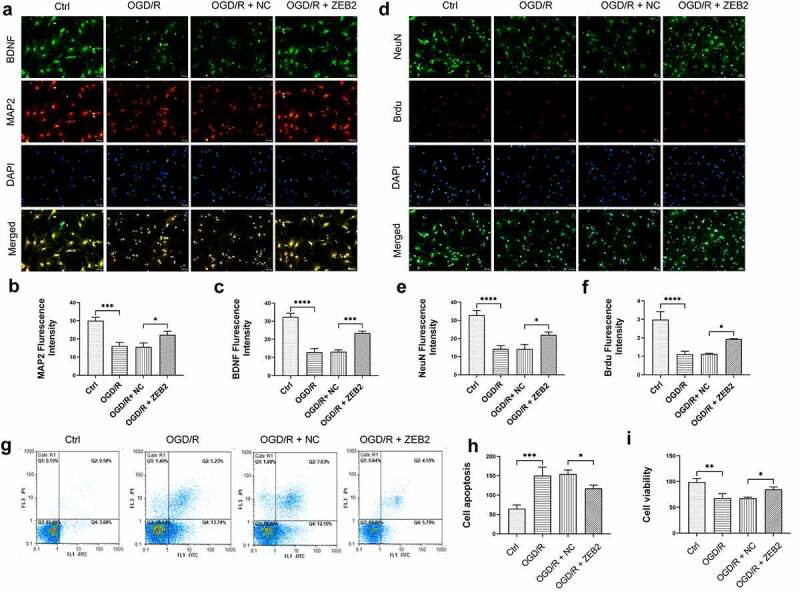
Astrogliosis promoted neuron regeneration *in vitro.* A: Immunofluorescence staining for NeuN+ (green fluorescence), BrdU+ (red fluorescence), and DAPI (blue fluorescence) in neurons. B: Immunofluorescence staining for BDNF (green fluorescence), MAP2+ (red fluorescence) and DAPI (blue fluorescence) in neurons. C: Fluorescence intensity of NeuN. D: Fluorescence intensity of BDNF. E: Fluorescence intensity of Brud. F: Fluorescence intensity of MAP2. G-H: Results of flow cytometry demonstrating astrogliosis alleviated apoptosis of neuron. I: Results of CCK8 demonstrating astrogliosis promoted proliferation of neuron (Error bars represent mean ± SD, Magnification:400×; *p < 0.05, **p < 0.01, ***p < 0.001, ****p < 0.0001)

To investigate the role of astrocytes-derived ZEB2 exerts in neuron regeneration, immunofluorescence assay was performed on co-cultured neurons. Brain-derived neurotrophic factor (BDNF) is a member of neurotrophic, it promoted new neurons growth and differentiation [50]. A specific marker of neuron Microtubule-associated proteins 2 (MAP2) was used to detect the number of neurons. The expression of BDNF and the number of neurons were both decreased under OGD/R condition, and overexpression of ZEB2 in astrocytes attenuated this trend (Figure 3a-c). Then, to detect the proliferation of neurons, the expression of Brdu in neurons was detected. Results demonstrated that the expression of Brdu, was decreased under OGD/R condition. While overexpression of ZEB2 in astrocytes reversed this trend, which indicated the increased proliferation of neurons (Figure 3d-f). In addition, proliferation and apoptosis of neuron was detected by CCK8 and flow cytometry, respectively. Results of CCK8 assay demonstrated that proliferation of neuron was decreased in OGD/R group compared with Ctrl group, while this trend alleviated by up-regulated expression of ZEB2 in astrocytes (Figure 3i). The apoptosis of neuron shown the opposite result (Figure 3g-h). These results indicated that, astrogliosis and neuron damage were observed under OGD/R condition, while up-regulated ZEB2 expression promoted a higher degree of astrogliosis, which promoted neuron regeneration.

**Figure 4.**
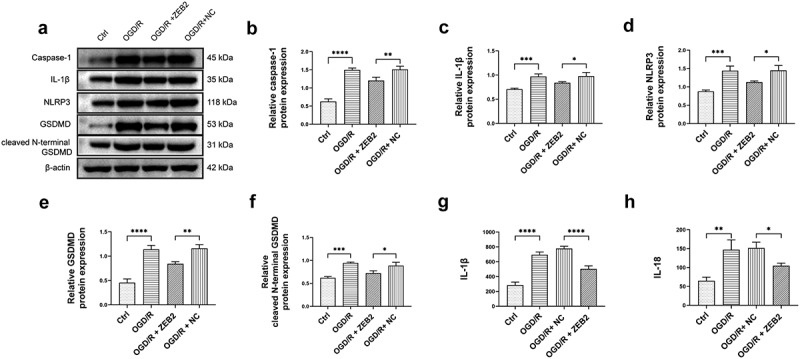
Astrogliosis promoted neuron regeneration by decreasing pyroptosis *in vitro.* A: Western blot assay of pyroptosis relative protein expression in neuron. B: Quantification of Caspase-1 Western blot band intensity. C: Quantification of IL-1β Western blot band intensity. D: Quantification of NLRP3 Western blot band intensity. E: Quantification of GSDMD Western blot band intensity. F: Quantification of cleaved N-terminal GSDMD Western blot band intensity. G: ELISA analysis of the secretion of IL-1β in neuron. H: ELISA analysis of the secretion of IL-18 in neuron. (Error bars represent mean ± SD; *p < 0.05, **p < 0.01, ***p < 0.001, ****p < 0.0001)

Previous studies reported that inflammasome-mediated pyroptosis play a key role in I/R injury of brain [51]. It is reasonable to assume that higher degree of astrogliosis induced by ZEB2 can protect neural regeneration from inflammation and pyroptosis. The results demonstrated that the expression of pyroptosis markers, i.e., caspase-1, IL-1β, NLRP3, GSDMD and cleaved N-terminal GSDMD was increased under OGD/R condition, and the overexpression of ZEB2 in astrocytes reversed the trend (Figure 4a-f). The results of ELISA shown that the secretion of IL-1β and IL-18 was increased under OGD/R condition, the overexpression of ZEB2 in astrocytes suppressed the secretion of these inflammatory cytokines (Figure 4g-h). Taken together, our results demonstrated that ZEB2 promoted astrogliosis, which protect neuron from pyroptosis under OGD/R condition.

### *ZEB2 promoted neural regeneration* in vivo

**Figure 5.**
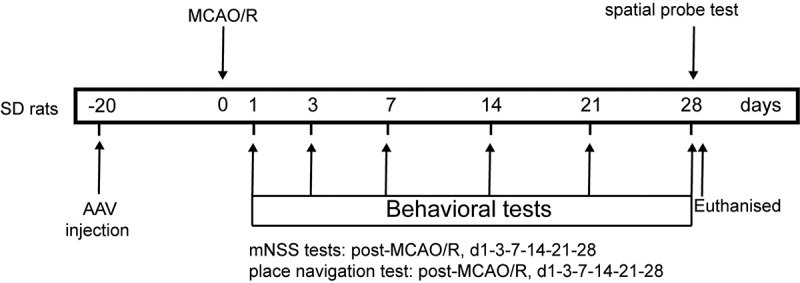
Timeline of experimental procedures (MCAO, reperfusion, behavioral tests, and brain harvested) in MCAO/R rats

**Figure 6.**
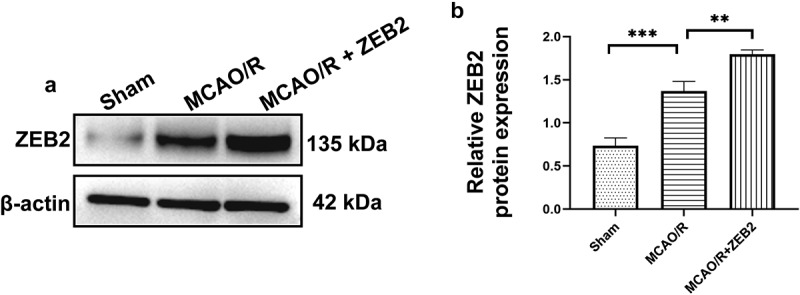
ZEB2 expression after AAV injection and MCAO/R model establishment. A. Western blot assay was used to evaluate the infection of AAV. B. Quantification of ZEB2 Western blot band intensity in each groups

**Figure 7.**
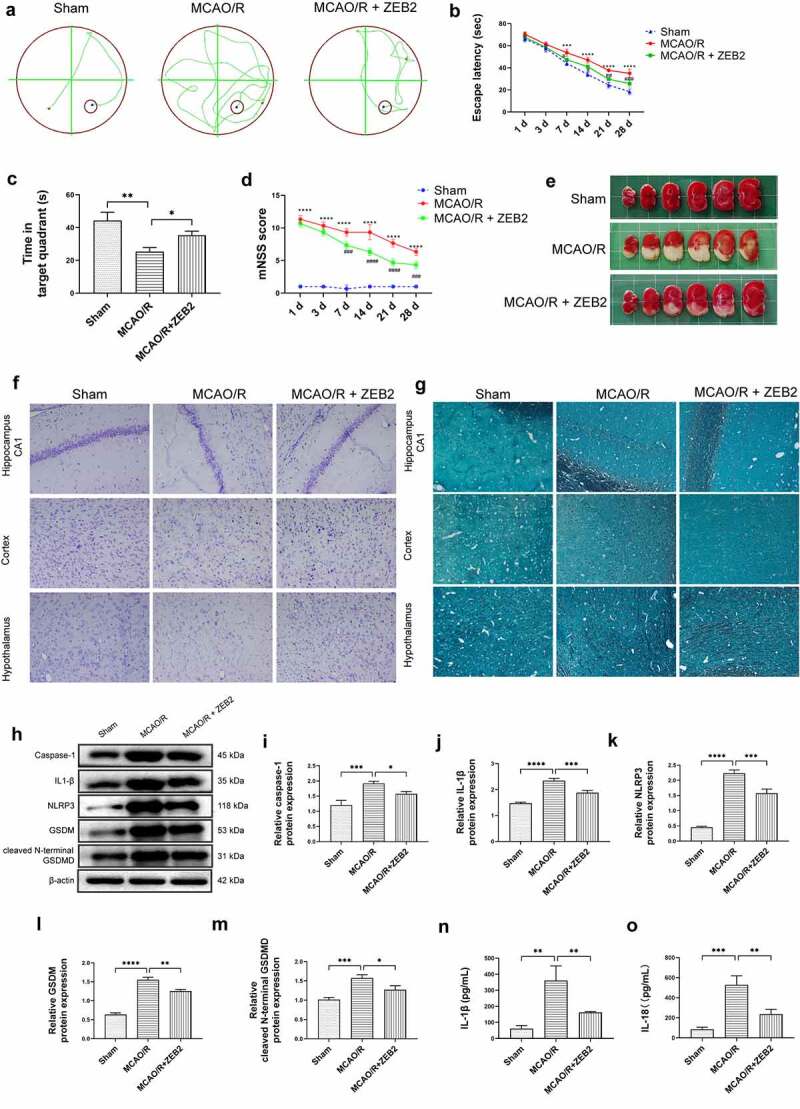
Overexpression of ZEB2 in brain tissues of MCAO/R rats promoted neuron regeneration by alleviating pyroptosis. A: Representative track images of each group mice. B: Mean escape latency time during the orientation navigation test on 1, 3,7,14,21,28 days after MCAO/R. C: Time of the rats stay in the target quadrant on 28 days after MCAO/R. D: mNSS score on 1, 3,7,14,21,28 days after MCAO/R. E: Representative images of brain slices stained by TTC in different groups at 28 days after MCAO/R. F: Staining by Nissl showed the Nissl bodies of hippocampus and cerebral cortex. G: Staining by Luxol fast blue (LFB) showed the myelin of hippocampus and cerebral cortex. H: Western blot analysis of the expression of pyroptosis protein i.e., caspase-1, IL-1β, NLRP3, gasdermin, cleaved N-terminal GSDMD in brain tissue. I: Relative protein expression of caspase-1. J. Relative protein expression of IL-1β. K: Relative protein expression of NLRP3. L: Relative protein expression of gasdermin, M: Relative protein expression of cleaved N-terminal GSDMD. N: ELISA analysis of the expression of IL-1β in brain tissue of rats on 28 days after MCAO/R. O: ELISA analysis of the expression of IL-18 in brain tissue of rats on 28 days after MCAO/R. (Error bars represent mean ± SD, Magnification:400×; *p < 0.05, **p < 0.01, ***p < 0.001, ****p < 0.0001

Then, we extended our work into an *in vivo* model. AAV-ZEB2 were injected into the brain at 20 days prior to middle cerebral artery occlusion/reperfusion (MCAO/R) model established, the experimental produces were shown in Figure 5. We first detected the expression of ZEB2 in brain tissue of rats in different groups, and found that ZEB2 was aberrantly overexpressed in MACO/R model. The injection of AAV further increased the expression of ZEB2 in brain tissue. Which suggested the successful infection of rAAV. (Figure 6a-b). Morris water maze test was performed to evaluate the spatial learning and memory capacity of rats. The MCAO/R rats needed longer time and more complicated route to find the hidden platform than sham group, and the overexpression of ZEB2 accelerated the time for MCAO/R rats finding the hidden platform, which also significantly simplified the route (Figure 7a-b). Moreover, the probe test shown the time in the target quadrant in MCAO/R rats were significantly decreased compared with Sham rats, and the overexpression of ZEB2 attenuated the trend (Figure 7c). Similarly, the score of the modified Neurological Severity Scores (mNSS) was significantly increased in MCAO/R group compared with sham group (Figure 7d). However, mNSS score was decreased when ZEB2 was overexpressed compared with MCAO/R group. TTC staining was used to detect the infarct area (Figure 7e). It indicated that the injection of AAV-ZEB2 alleviated the tissue loss caused by ischemia. These results suggested that MCAO/R rats had a neurological deficit, and ZEB2 overexpression alleviated this deficit. Nissl body is a large granular body in neuron, which is a kind of rough endoplasmic reticulum with rosettes of free ribosomes, and is the site of protein synthesis [52]. Nissl body decreased in injury neuron [53]. The results of Nissl staining (figure 7f) indicated that the number of Nissl bodies was decreased in cerebral cortex, hypothalamus and hippocampus in MCAO/R rats compared with sham rats, while ZEB2 reversed the result. The above experiments demonstrated that up-regulated ZEB2 promoted neuron regeneration after MCAO/R injury. To detect the role of ZEB2 on the neuronal damage, Luxol Fast Blue (LFB) staining were performed to observe the myelin and Bielschowsky sliver staining was used to observe axons. The number of myelin and axon was significant decreased in cerebral cortex, hippocampus and hypothalamus of MCAO/R rats compared with sham rats, and then ZEB2 reversed the result (Figure 7g). Also, demyelination in nuclei observed in MCAO/R rats was also alleviated by ZEB2 overexpression. To further investigated the mechanism of ZEB2 attenuated neuron damage in MCAO/R rats, the level of inflammasome and pyroptosis was detected by ELISA and Western blot. (Figure 7h-o). We found upregulated ZEB2 alleviate the protein expression of pyroptosis markers, i.e., caspase-1, IL-1β, IL-18, NLRP3, GSDMD and cleaved N-terminal GSDMD. Taken together, our results demonstrated that ZEB2 promoted neural regeneration of brain in MCAO/R rats by decreased the inflammation and pyroptosis.

### Discussion

Cerebral ischemia causes irreversible cell death or impairment of neuron and astrocytes, which therefore lead to neuronal dysfunction [54]. Recently, amounts of studies found that brain can participate in its functional recovery in early stage after stroke onset. Several evidences shown that ZEB2 plays a key role in neural regeneration, including in ischemia stroke [29,35]. Nevertheless, the relationship between the expression of ZEB2 and neural regeneration in cerebral ischemia stroke still unknow. In this study, we investigated the potential effect of ZEB2 in promoting astrocyte proliferation and therefore provide a favorable environment for nerve restoration by protecting neurons adjacent to lesion from pyroptosis.

ZEB2 (also known as Sip1 or Zfhx1b), along with ZEB1 (also known as dEF1 or Zfhx1a), is belongs to ZEB family of two-handed zinc finger/homeodomain protein. It is acknowledged that, ZEB2 involves in various process of generation and development of the nervous systems including formation, delamination, migration, and specification of neural crest cells, as well as the development of various neuronal and glial populations [35,55]. However, the pervious result showed that ZEB2 is absent in neurons [28]. Astrogliosis is a process of abnormal astrocytes activity accompanied with cellular hypertrophy, cellular thickened and up-regulation of glial fibrillary acidic protein (GFAP) [56]. Severe astrogliosis form an astrocyte scar, which are a physical barrier isolates the lesion and protects surrounding tissue from further damage and promoted axon regeneration [28,57]. In addition, astrocyte scars also inhibited neuron regeneration by absorbs harmful injury factors and secretes neurotrophic factors [58]. It is therefore notable in our result that ZEB2 can further increase the expression of GFAP, a hallmark signal for activation of astrocyte. This means ZEB2 can inducted reactive astrocytes, which is consisted with pervious result [28].

A major cause of blood-brain barrier damage and brain injury in ischemic diseases is thought to be inflammation caused by microglia or necrotic cells [59]. Pyroptosis is a type of programmed cell death that is responsive to inflammation [13]. Inflammasomes are multiple protein complex that form when stimulated by exogenous microbial invasion and endogenous damage signals [60]. When stimulation occurs, inflammasomes formed in the cytoplasm via the classical pyroptosis pathway, which is dependent on caspase 1, or/ and the non-classical pyroptosis pathway, which is dependent on caspase 4/5/11 [51]. The non-classical pathway of pyroptosis is mainly involved in infectious inflammatory diseases, whereas the classical pathway of pyroptosis is mainly associated with noninfectious inflammatory diseases, which includes ischemia injury [51,61]. NLRP3 inflammasome is widely observed in cerebral ischemia tissues and reported to play a significant role in CNS injury in ischemic diseases [62]. Inhibition of NLRP3-inflammasome is reported to alleviated the brain injury in ischemia [61]. To verify the mechanism, we co-cultured astrocytes and neurons. We found co-cultured astrocytes can promote the expression of BDNF and proliferation of neuron, and inhibit the cell apoptosis induced by ischemia. Also, ZEB2 overexpression *in vivo* significantly recovered neural injury caused by ischemia. Brain-derived neurotrophic factor (BDNF) is a member of neurotrophic, it promoted new neurons growth and differentiation [63]. Studies proved that an aberrant activation of astrocytes can secret neuroprotective cytokines, and therefore promote the neural regeneration [19,28]. Which is consist with our results. Also, our results indicated that overexpression of ZEB2 in astrocytes decreased the expression of pyroptosis associated genes. We therefore conclude from the above results that ZEB2 promoted astrogliosis, an aberrant activation of astrocytes, therefore enhances neural regeneration by decreasing the inflammation and pyroptosis.

### Conclusion

Our findings demonstrated that ZEB2 promoted astrogliosis, which therefore enhances neural regeneration by decreasing the inflammation and pyroptosis. It offered a new potential therapeutic method for cerebral I/R injury.

## Data Availability

All data and materials are available from the corresponding author on reasonable request.

## References

[1] Wang W, Jiang, B, Sun, H, et al. NESS-China Investigators. Prevalence, Incidence, and Mortality of Stroke in China: Results from a Nationwide Population-Based Survey of 480 687 Adults. Circulation. 2017 Feb;135(8):759–771. doi:10.1161/CIRCULATIONAHA.116.025250. 28052979

[2] Campbell BCV, De Silva, DA, Macleod, MR, et al. Ischaemic stroke. Nat Rev Dis Primers. 2019 Oct 10;5(1):70. doi:10.1038/s41572-019-0118-8. 31601801

[3] Kerendi F, Kin H, Halkos ME, et al. Remote postconditioning. brief renal ischemia and reperfusion applied before coronary artery reperfusion reduces myocardial infarct size via endogenous activation of adenosine receptors. Basic Res Cardiol. 2005;100(5):404–412. 15965583 10.1007/s00395-005-0539-2

[4] Kamel H, Healey JS. Cardioembolic Stroke. Circ Res. 2017;120(3):514–526. 28154101 10.1161/CIRCRESAHA.116.308407 PMC5312810

[5] Suzuki K, Matsumaru, Y, Takeuchi, M, et al. Effect of mechanical thrombectomy without vs with intravenous thrombolysis on functional outcome among patients with acute ischemic stroke: the SKIP randomized clinical trial. JAMA. 2021 Jan 19;325(3):244–253. doi:10.1001/jama.2020.23522. 33464334 PMC7816103

[6] Jean WC, Spellman SR, Nussbaum ES, et al. Reperfusion injury after focal cerebral ischemia: the role of inflammation and the therapeutic horizon. Neurosurgery. 1998;43(6):1382–1396. discussion 1396-7. 9848853 10.1097/00006123-199812000-00076

[7] Stocchetti N, Taccone FS, Citerio G, et al. Neuroprotection in acute brain injury: an up-to-date review. Crit Care. 2015;19(1):186. 25896893 10.1186/s13054-015-0887-8 PMC4404577

[8] Sha R, Zhang B, Han X, et al. Electroacupuncture alleviates ischemic brain injury by inhibiting the miR-223/NLRP3 pathway. Med Sci Monit. 2019;25:4723–4733. 31237865 10.12659/MSM.917213 PMC6607941

[9] Srivastava P, Cronin CG, Scranton VL, et al. Neuroprotective and neuro-rehabilitative effects of acute purinergic receptor P2X4 (P2X4R) blockade after ischemic stroke. Exp Neurol. 2020;329:113308. 32289314 10.1016/j.expneurol.2020.113308 PMC7242087

[10] Matei N, Camara, J, McBride, D, et al. Intranasal wnt3a attenuates neuronal apoptosis through Frz1/PIWIL1a/FOXM1 pathway in MCAO rats. J Neurosci. 2018 Jul 25;38(30):6787–6801. doi:10.1523/JNEUROSCI.2352-17.2018. 29954850 PMC6067074

[11] Liang J, Wang Q, Li J-Q, et al. Long non-coding RNA MEG3 promotes cerebral ischemia-reperfusion injury through increasing pyroptosis by targeting miR-485/AIM2 axis. Exp Neurol. 2020;325:113139. 31794744 10.1016/j.expneurol.2019.113139

[12] Zhou Y, Gu Y, Liu J. BRD4 suppression alleviates cerebral ischemia-induced brain injury by blocking glial activation via the inhibition of inflammatory response and pyroptosis. Biochem Biophys Res Commun. 2019;519(3):481–488. 31530390 10.1016/j.bbrc.2019.07.097

[13] Frank D, Vince JE. Pyroptosis versus necroptosis: similarities, differences, and crosstalk. Cell Death Differ. 2019;26(1):99–114. 30341423 10.1038/s41418-018-0212-6 PMC6294779

[14] Tang R, Xu J, Zhang B, et al. Ferroptosis, necroptosis, and pyroptosis in anticancer immunity. J Hematol Oncol. 2020;13(1):110. 32778143 10.1186/s13045-020-00946-7 PMC7418434

[15] Hu L, Chen, M, Chen, X, et al. Chemotherapy-induced pyroptosis is mediated by BAK/BAX-caspase-3-GSDME pathway and inhibited by 2-bromopalmitate. Cell Death Dis. 2020 Apr 24;11(4):281. doi:10.1038/s41419-020-2476-2. 32332857 PMC7181755

[16] Sun L, Ma W, Gao W, et al. Propofol directly induces caspase-1-dependent macrophage pyroptosis through the NLRP3-ASC inflammasome. Cell Death Dis. 2019;10(8):542. 31316052 10.1038/s41419-019-1761-4 PMC6637184

[17] Poh L, Kang S-W, Baik S-H, et al. Evidence that NLRC4 inflammasome mediates apoptotic and pyroptotic microglial death following ischemic stroke. Brain Behav Immun. 2019;75:34–47. 30195027 10.1016/j.bbi.2018.09.001

[18] Liang YB, Song -P-P, Zhu Y-H, et al. TREM-1-targeting LP17 attenuates cerebral ischemia-induced neuronal injury by inhibiting oxidative stress and pyroptosis. Biochem Biophys Res Commun. 2020;529(3):554–561. 32736673 10.1016/j.bbrc.2020.05.056

[19] Guillamón-Vivancos T, Gómez-Pinedo U, Matías-Guiu J. Astrocytes in neurodegenerative diseases (I): function and molecular description. Neurologia. 2015;30(2):119–129. 23465689 10.1016/j.nrl.2012.12.007

[20] Liu H, Wu X, Luo J, et al. pterostilbene attenuates astrocytic inflammation and neuronal oxidative injury after ischemia-reperfusion by inhibiting NF-κB phosphorylation. Front Immunol. 2019;10:2408. 31681297 10.3389/fimmu.2019.02408 PMC6811521

[21] Durkee CA, Araque A. Diversity and specificity of astrocyte-neuron communication. Neuroscience. 2019;396:73–78. 30458223 10.1016/j.neuroscience.2018.11.010 PMC6494094

[22] Sofroniew MV. Molecular dissection of reactive astrogliosis and glial scar formation. Trends Neurosci. 2009;32(12):638–647. 19782411 10.1016/j.tins.2009.08.002 PMC2787735

[23] Li X, Li M, Tian L, et al. Reactive astrogliosis: implications in spinal cord injury progression and therapy. Oxid Med Cell Longev. 2020;2020:9494352. 32884625 10.1155/2020/9494352 PMC7455824

[24] Zhang D, Hu X, Qian L, et al. Astrogliosis in CNS pathologies: is there a role for microglia? Mol Neurobiol. 2010;41(2–3):232–241. 20148316 10.1007/s12035-010-8098-4 PMC3629545

[25] Anderson MA, Burda JE, Ren Y, et al. Astrocyte scar formation aids central nervous system axon regeneration. Nature. 2016;532(7598):195–200. 27027288 10.1038/nature17623 PMC5243141

[26] Wanner IB, Anderson MA, Song B, et al. Glial scar borders are formed by newly proliferated, elongated astrocytes that interact to corral inflammatory and fibrotic cells via STAT3-dependent mechanisms after spinal cord injury. J Neurosci. 2013;33(31):12870–12886. 23904622 10.1523/JNEUROSCI.2121-13.2013 PMC3728693

[27] Ellison JA, Velier JJ, Spera P, et al. Osteopontin and its integrin receptor αvβ3 are upregulated during formation of the glial scar after focal stroke. Stroke. 1998;29(8):1698–1706. discussion 1707. 9707214 10.1161/01.str.29.8.1698

[28] Vivinetto AL, Kim, ID, Goldberg, DC, et al. Zeb2 is a regulator of astrogliosis and functional recovery after CNS injury. Cell Rep. 2020 Jun 30;31(13):107834. doi:10.1016/j.celrep.2020.107834. 32610135 PMC7416489

[29] Epifanova E, Babaev A, Newman AG, et al. Role of Zeb2/Sip1 in neuronal development. Brain Res. 2019;1705:24–31. 30266271 10.1016/j.brainres.2018.09.034

[30] Sun X, Hu, X, Wang, D, et al. Establishment and characterization of primary astrocyte culture from adult mouse brain. Brain Res Bull. 2017 Jun;132:10–19. doi:10.1016/j.brainresbull.2017.05.002. 28499803

[31] Li H, Mar BG, Zhang H, et al. The EMT regulator ZEB2 is a novel dependency of human and murine acute myeloid leukemia. Blood. 2017;129(4):497–508. 27756750 10.1182/blood-2016-05-714493 PMC5270388

[32] Jiang Y, Liu G, Ye W, et al. ZEB2-AS1 accelerates epithelial/mesenchymal transition through miR-1205/CRKL pathway in colorectal cancer. Cancer Biother Radiopharm. 2020;35(2):153–162. 31755734 10.1089/cbr.2019.3000

[33] Zhang J, Zhang H, Qin Y, et al. MicroRNA-200c-3p/ZEB2 loop plays a crucial role in the tumor progression of prostate carcinoma. Ann Transl Med. 2019;7(7):141. 31157262 10.21037/atm.2019.02.40 PMC6511553

[34] Suh HN, Han HJ. Sonic hedgehog increases the skin wound-healing ability of mouse embryonic stem cells through the microRNA 200 family. Br J Pharmacol. 2015;172(3):815–828. 25257936 10.1111/bph.12947 PMC4301691

[35] Hegarty SV, Sullivan AM, O’Keeffe GW. Zeb2: a multifunctional regulator of nervous system development. Prog Neurobiol. 2015;132:81–95. 26193487 10.1016/j.pneurobio.2015.07.001

[36] Ioannou MS, Jackson J, Sheu S-H, et al. Neuron-Astrocyte metabolic coupling protects against activity-induced fatty acid toxicity. Cell. 2019;177(6):1522–1535.e14. 31130380 10.1016/j.cell.2019.04.001

[37] Beaudoin GM 3rd, Lee S-H, Singh D, et al. Culturing pyramidal neurons from the early postnatal mouse hippocampus and cortex. Nat Protoc. 2012;7(9):1741–1754. 22936216 10.1038/nprot.2012.099

[38] Zoghebi KA, Bousoik E, Parang K, et al. Design and biological evaluation of colchicine-CD44-targeted peptide conjugate in an in vitro model of crystal induced inflammation. Molecules. 2019;25(1):46. 10.3390/molecules25010046 PMC6982808 31877739

[39] Kallubai M, Reddy SP, Dubey S, et al. Spectroscopic evaluation of synthesized 5β-dihydrocortisol and 5β-dihydrocortisol acetate binding mechanism with human serum albumin and their role in anticancer activity. J Biomol Struct Dyn. 2019;37(3):623–640. 29375009 10.1080/07391102.2018.1433554

[40] Hammond SL, Leek AN, Richman EH, et al. Cellular selectivity of AAV serotypes for gene delivery in neurons and astrocytes by neonatal intracerebroventricular injection. PLoS One. 2017;12(12):e0188830. 29244806 10.1371/journal.pone.0188830 PMC5731760

[41] Longa EZ, Weinstein PR, Carlson S, et al. Reversible middle cerebral artery occlusion without craniectomy in rats. Stroke. 1989;20(1):84–91. 2643202 10.1161/01.str.20.1.84

[42] Nagasawa H, Kogure K. Correlation between cerebral blood flow and histologic changes in a new rat model of middle cerebral artery occlusion. Stroke. 1989;20(8):1037–1043. 2756535 10.1161/01.str.20.8.1037

[43] Chen J, Sanberg PR, Li Y, et al. Intravenous administration of human umbilical cord blood reduces behavioral deficits after stroke in rats. Stroke. 2001;32(11):2682–2688. 11692034 10.1161/hs1101.098367

[44] Gallagher M, Burwell R, Burchinal M. Severity of spatial learning impairment in aging: development of a learning index for performance in the morris water maze. Behav Neurosci. 2015;129(4):540–548. 26214219 10.1037/bne0000080 PMC5640430

[45] Fan H, Tang H-B, Chen Z, et al. Inhibiting HMGB1-RAGE axis prevents pro-inflammatory macrophages/microglia polarization and affords neuroprotection after spinal cord injury. J Neuroinflammation. 2020;17(1):295. 33036632 10.1186/s12974-020-01973-4 PMC7547440

[46] Yang L, Liu Y, Wang Y, et al. Azeliragon ameliorates alzheimer’s disease via the janus tyrosine kinase and signal transducer and activator of transcription signaling pathway. Clinics (Sao Paulo). 2021;76:e2348. 33681944 10.6061/clinics/2021/e2348 PMC7920406

[47] Benedek A, Móricz K, Jurányi Z, et al. Use of TTC staining for the evaluation of tissue injury in the early phases of reperfusion after focal cerebral ischemia in rats. Brain Res. 2006;1116(1):159–165. 16952339 10.1016/j.brainres.2006.07.123

[48] Li N, Liu C, Wang C, et al. Early changes of NLRP3 inflammasome activation after hypoxic-ischemic brain injury in neonatal rats. Int J Clin Exp Pathol. 2021;14(2):209–220. 33564353 PMC7868790

[49] Yang Z, Wang KK. Glial fibrillary acidic protein: from intermediate filament assembly and gliosis to neurobiomarker. Trends Neurosci. 2015;38(6):364–374. 25975510 10.1016/j.tins.2015.04.003 PMC4559283

[50] Ward R, Li W, Abdul Y, et al. NLRP3 inflammasome inhibition with MCC950 improves diabetes-mediated cognitive impairment and vasoneuronal remodeling after ischemia. Pharmacol Res. 2019;142:237–250. 30818045 10.1016/j.phrs.2019.01.035 PMC6486792

[51] Chen XX, Qian Y, Wang X-P, et al. Nurr1 promotes neurogenesis of dopaminergic neuron and represses inflammatory factors in the transwell coculture system of neural stem cells and microglia. CNS Neurosci Ther. 2018;24(9):790–800. 29450981 10.1111/cns.12825 PMC6489950

[52] Li J, Wen P-Y, Li -W-W, et al. Upregulation effects of tanshinone IIA on the expressions of neun, nissl body, and IκB and downregulation effects on the expressions of GFAP and NF-κB in the brain tissues of rat models of alzheimer’s disease. Neuroreport. 2015;26(13):758–766. 26164608 10.1097/WNR.0000000000000419

[53] Johnson IP, Pullen AH, Sears TA. Target dependence of nissl body ultrastructure in cat thoracic motoneurones. Neurosci Lett. 1985;61(1–2):201–205. 4080255 10.1016/0304-3940(85)90425-2

[54] Pekny M, Nilsson M. Astrocyte activation and reactive gliosis. Glia. 2005;50(4):427–434. 15846805 10.1002/glia.20207

[55] Quintes S, Brinkmann BG, Ebert M, et al. Zeb2 is essential for schwann cell differentiation, myelination and nerve repair. Nat Neurosci. 2016;19(8):1050–1059. 27294512 10.1038/nn.4321 PMC4964942

[56] Sofroniew MV. Astrogliosis. Cold Spring Harb Perspect Biol. 2014;7(2):a020420. 25380660 10.1101/cshperspect.a020420 PMC4315924

[57] Trendelenburg G, Dirnagl U. Neuroprotective role of astrocytes in cerebral ischemia: focus on ischemic preconditioning. Glia. 2005;50(4):307–320. 15846804 10.1002/glia.20204

[58] Sofroniew MV. Astrocyte barriers to neurotoxic inflammation. Nat Rev Neurosci. 2015;16(5):249–263. 25891508 10.1038/nrn3898 PMC5253239

[59] Huang L, Chen C, Zhang X, et al. Neuroprotective effect of curcumin against cerebral Ischemia-Reperfusion via mediating autophagy and inflammation. J Mol Neurosci. 2018;64(1):129–139. 29243061 10.1007/s12031-017-1006-x

[60] Sharif H, Wang L, Wang WL, et al. Structural mechanism for NEK7-licensed activation of NLRP3 inflammasome. Nature. 2019;570(7761):338–343. 31189953 10.1038/s41586-019-1295-z PMC6774351

[61] Ismael S, Zhao L, Nasoohi S, et al. Inhibition of the NLRP3-inflammasome as a potential approach for neuroprotection after stroke. Sci Rep. 2018;8(1):5971. 29654318 10.1038/s41598-018-24350-x PMC5899150

[62] An P, Xie J, Qiu S, et al. Hispidulin exhibits neuroprotective activities against cerebral ischemia reperfusion injury through suppressing NLRP3-mediated pyroptosis. Life Sci. 2019;232:116599. 31247210 10.1016/j.lfs.2019.116599

[63] Song M, Martinowich K, Lee FS. BDNF at the synapse: why location matters. Mol Psychiatry. 2017;22(10):1370–1375. 28937692 10.1038/mp.2017.144 PMC5646361

